# Insertion of an Esterase Gene into a Specific Locust Pathogen (*Metarhizium acridum*) Enables It to Infect Caterpillars

**DOI:** 10.1371/journal.ppat.1002097

**Published:** 2011-06-23

**Authors:** Sibao Wang, Weiguo Fang, Chengshu Wang, Raymond J. St. Leger

**Affiliations:** 1 Department of Entomology, University of Maryland, College Park, Maryland, United States of America; 2 Institute of Plant Physiology and Ecology, Shanghai Institutes for Biological Sciences, Chinese Academy of Sciences, Shanghai, China; University of Melbourne, Australia

## Abstract

An enduring theme in pathogenic microbiology is poor understanding of the mechanisms of host specificity. *Metarhizium* is a cosmopolitan genus of invertebrate pathogens that contains generalist species with broad host ranges such as *M. robertsii* (formerly known as *M. anisopliae* var. *anisopliae*) as well as specialists such as the acridid-specific grasshopper pathogen *M. acridum*. During growth on caterpillar (*Manduca sexta*) cuticle, *M. robertsii* up-regulates a gene (*Mest1*) that is absent in *M. acridum* and most other fungi. Disrupting *M. robertsii Mest1* reduced virulence and overexpression increased virulence to caterpillars (*Galleria mellonella* and *M. sexta*), while virulence to grasshoppers (*Melanoplus femurrubrum*) was unaffected. When *Mest1* was transferred to *M. acridum* under control of its native *M. robertsii* promoter, the transformants killed and colonized caterpillars in a similar fashion to *M. robertsii*. MEST1 localized exclusively to lipid droplets in *M. robertsii* conidia and infection structures was up-regulated during nutrient deprivation and had esterase activity against lipids with short chain fatty acids. The mobilization of stored lipids was delayed in the *Mest1* disruptant mutant. Overall, our results suggest that expression of *Mest1* allows rapid hydrolysis of stored lipids, and promotes germination and infection structure formation by *M. robertsii* during nutrient deprivation and invasion, while *Mest1* expression in *M. acridum* broadens its host range by bypassing the regulatory signals found on natural hosts that trigger the mobilization of endogenous nutrient reserves. This study suggests that speciation in an insect pathogen could potentially be driven by host shifts resulting from changes in a single gene.

## Author Summary

Host selectivity and host switching have been widely documented in diverse pathogens, but in most cases the underlying mechanisms are poorly understood. Entomopathogenic fungi in the genus *Metarhizium* are being used as environmentally friendly alternatives to chemical insecticides in agricultural and human disease-vector control programs, and as model systems for studying the interactions between invertebrate hosts and pathogenic fungi. In this paper we describe molecular mechanisms controlling the host selectivity of *M. robertsii* strain Mr2575, a generalist able to infect hundreds of insect species, and *M. acridum* strain Ma324, a specialist pathogen of grasshoppers and locusts. The esterase gene (*Mest1*) from Mr2575 is required for virulence against caterpillars but not grasshoppers. Ma324 lacks *Mest1*, but insertion of *Mest1* into Ma324 broadens its host range to include caterpillars. Our results suggest that expression of *Mest1* allows rapid mobilization of endogenous lipid reserves and promotes germination and infection structure formation. This study suggests that speciation in insect pathogens can be driven by host shifts due to the gain or loss of a pathogen gene that confers wide host specificity.

## Introduction

An enduring theme in pathogenic microbiology is poor understanding of the mechanisms of host specificity. That is, what factors limit a pathogens host range, how is host specificity linked with virulence and what changes in pathogens or hosts can open new host ranges? These are fundamental questions that relate both to the co-evolution of host susceptibility and pathogen virulence, as well as to factors underlying host switching and the emergence of new pathogens that originate in different host species. The molecular mechanisms controlling host selectivity in fungi are particularly poorly understood. A number of plant pathogenic fungi produce secondary metabolites with biological specificities that correspond with the host range of the producing fungi [1], [2], and small secreted proteins produced by the pathogen can trigger resistance in some plants limiting host range [3]. However, to date these have not been found in animal pathogenic fungi, many of which seem to be broadly opportunistic. This has allowed researchers on emergent human pathogenic fungi to employ insects as model systems [4]. Most human pathogens are not normally transmitted between insect hosts. However, specialization to entomopathogenicity is a major fungal lifestyle with ∼1000 known species that can be highly infectious. In contrast to the opportunistic pathogens, many of these have evolved narrow host ranges by as yet unknown mechanisms.

*Metarhizium* is a cosmopolitan genus of Ascomycetes (class Sordariomycetes) comprising species that exhibit varied lifestyles. *Metarhizium robertsii* (formerly known as *M. anisopliae* var. *anisopliae* [5]) is a generalist able to infect hundreds of insect species. It has been at the forefront of efforts to develop biocontrol alternatives to chemical insecticides in agricultural and human disease-vector control programs [6]–[8]. *M. robertsii* has also been used to study the interactions between invertebrate model hosts and pathogenic fungi as host innate immune responses are broadly conserved across many phyla [9]. In contrast *M. acridum* is a specialist with a narrow host range for certain locusts and grasshoppers [10]. This specificity is one of the reasons it is being mass produced as an environmentally safe alternative to pesticides [10]–[12].

The infection strategies of *Metarhizium* species resemble those of most plant pathogens. Infection proceeds via spores that adhere to the host surface and germinate to form a germ tube that continues undifferentiated hyphal growth if nutrient quality and quantity are not conducive for differentiation of infection structures. On a host, however, apical elongation terminates and germ tubes produce infection structures, called appressoria, which promote the localized production of cuticle degrading enzymes and also build up turgor providing a mechanical component to penetration [13]–[15]. The formation of appressoria by broad host range strains of *M. robertsii* such as ARSEF2575 (Mr2575) can also be induced efficiently by low levels of complex nitrogenous nutrients [13]. However, pathogens with a narrow host range such as *M. acridum* ARSEF324 (Ma324) ( = CSIRO FI 485: the active ingredient of ‘Green Guard’) germinate poorly under these conditions and only produce abundant appressoria in the presence of a lipid extract from host insects [16].

*M. robertsii* Mr2575 sharply up-regulates the *Mest1* gene when germinating on insect cuticles [17]. This gene is absent in Ma324 [18], which suggests that it is unlikely to have an essential function in the related *M. robertsii* but we speculated that it might have a niche role in pathogenicity, perhaps facilitating an opportunistic life-style. In this study, we functionally characterized MEST1 and demonstrated that inserting *Mest1* into *M. acridum* is sufficient to expand its host range to include lepidopterans. *Mest1* is thus the first gene identified in an entomopathogenic fungus that encodes a determinant of specificity and is to our knowledge the first example where a single metabolic protein assumes such a crucial role for host selectivity in a animal pathogenic fungus. We speculate that speciation in insect pathogens can be driven by host shifts that become fixed in populations due to the gain or loss of a pathogen gene that confers wide host specificity.

## Results

### Gene cloning, molecular characterization and gene disruption of *Mest1*

The *M. robertsii Mest1* is 1188 bp long and lacks introns. It encodes a predicted protein of 395 amino acids, with a deduced molecular weight of 42244 Da and a pI of 5.34. The SignalP 3.0 program (http://www.cbs.dtu.dk/services/SignalP/) revealed no signal sequence suggesting that MEST1 is a cell-bound protein. MEST1 contains the sequence GGS^341^VG which conforms to the motif G-X1-S-X2-G, commonly observed in serine esterases and many lipases [19]–[21]. According to *M. robertsii* genome sequence data [22], *Mest1* (MAA_03283) is downstream of three secreted cuticle degrading subtilisins (Pr1F (MAA_03280), Pr1E (MAA_03281) and a subtilisin-like protease (MAA_03282). The *M. robertsii* genome also contains a paralog (MAA_08059) with 42% identity to MEST1 that is downstream of three hypothetical proteins and upstream of a glutathione S-transferase. The genome of *M. acridum* strain CQMa 102 lacks an ortholog of MEST1 but contains a sequence (MAC_02852) with 90.2% identity to MAA_08059 (the median sequence identity of orthologs is 89.8%). MAC_02852 is downstream of four hypothetical proteins and upstream of glutathione S-transferase indicating that it is syntenic with its ortholog MAA_08059.

**Figure 1:**
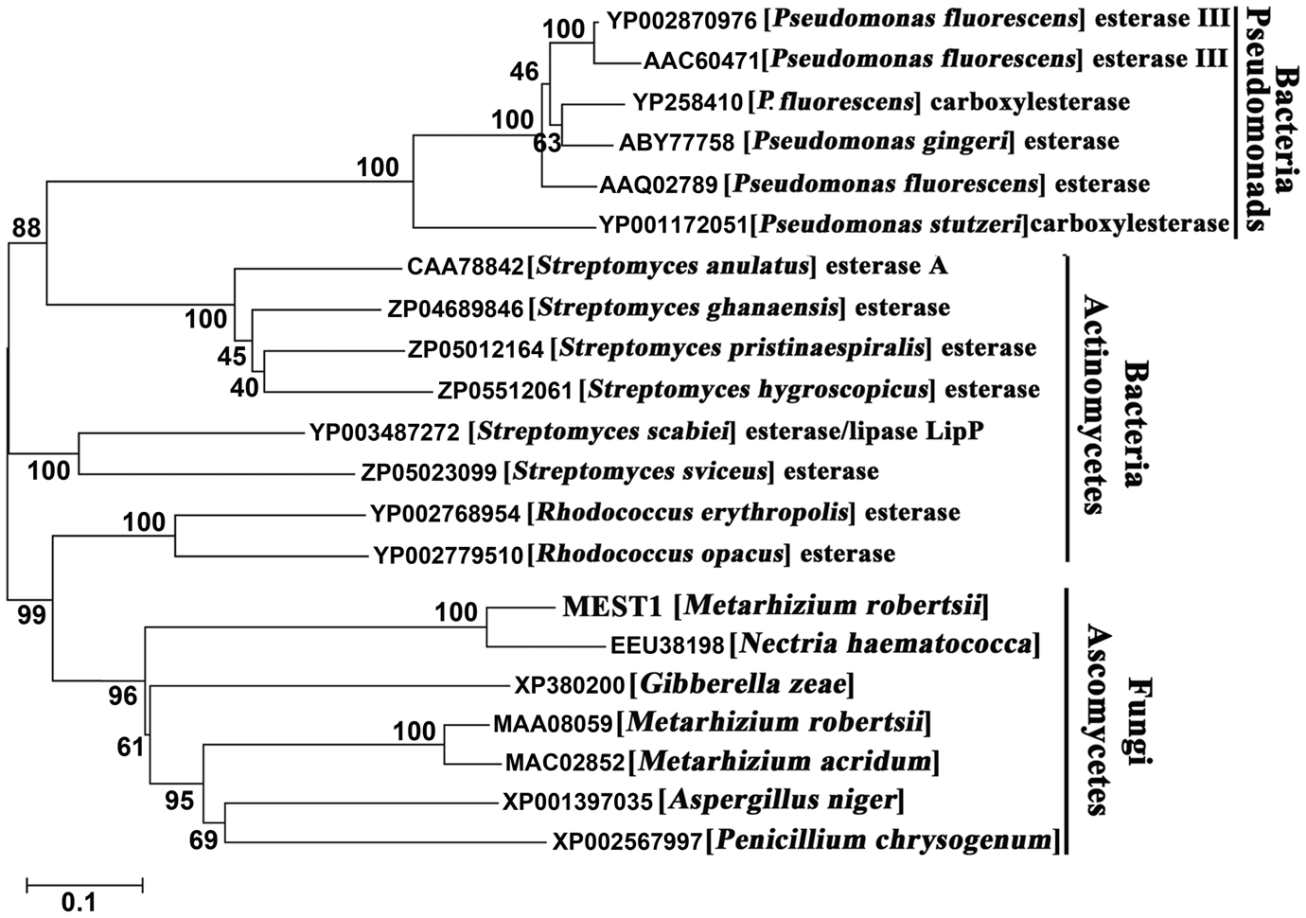
Phylogenetic relationship of MEST1 with its homologs. The amino acid sequences were aligned with Clustal X and a neighbor-joining tree was generated with 1,000 boot strap replicates using the program MEGA v4.0.

Homologs to MEST1 and MAA_08059 were identified in four other ascomycete fungi, but only the related *Nectria haematococca* had a sequence (EEU_38198) that was highly similar (82% identity) to MEST1. The related *Gibberella zeae* (XP_380200) as well as *Aspergillus niger* (XP_001397035) and *Penicillium chrysogenum* (XP_002567997) contain single copy sequences resembling MAA_08059 (Figure 1). The functions of these genes have not been reported and contain a putative penicillin-binding domain characteristic of β-lactamase class C proteins. Homologs of MEST1 were absent in *Neurospora crassa*, *Magnaporthe grisea*, *Schizosaccharomyces japonicus*, *Trichoderma harzianum* and *T. reesei*, as well as Basidiomycetes, Zygomycetes and Chytridiomycetes. However, MEST1 shows up to 42% identity with bacterial sequences, including known esterases [19]. A phylogenetic tree (Figure 1) confirmed that MEST1 and the fungal homologs formed a separate well supported clade distinct from bacterial clades containing actinomycete and pseudomonad sequences.

### MEST1 regulates virulence and appressorial differentiation in *Metarhizium robertsii*

**Figure 2:**
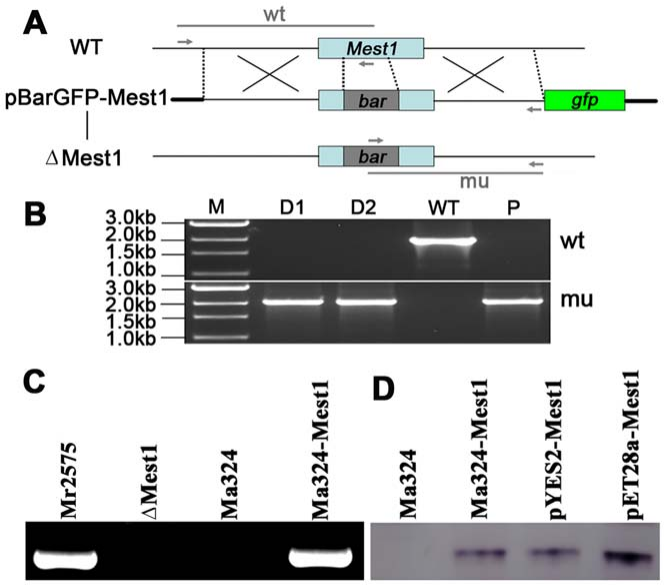
Disruption of the *Mest1* gene in *Metarhizium robertsii* Mr2575. (**A**) Schematic representation of the *Mest1* wild type (WT) locus and the plasmid pBarGFP-Mest1 (containing two regions homologous to the *Mest1* reading frame), that was used for gene disruption through double crossover recombination. Replacement- and WT-specific primer combinations and expected fragments are shown as grey lines. (**B**) Replacement-specific PCR analysis. Confirmation of the predicted gene targeting conducted with primer combinations that only amplify a signal in the recombinant locus (mu). The absence of a WT-specific signal in the clonal disruptants ΔMest1 (D1, D2) and plasmid pBarGFP-Mest1 (P) confirms the genetic homogeneity of the mutant isolates. (**C**) MEST1 expression in *M. acridum* Ma324 was verified by RT-PCR using cDNA from wild type Mr2575, Ma324, mutant (ΔMest1) and transgenic strain Ma324-Mest1. (**D**) MEST1 expression in *M. acridum* Ma324-Mest1, *E. coli* (pYES2-Mest1), and yeast cells (pET28a-Mest1) was confirmed by Western blot using a 6-Histidine Epitope Tag Antibody.

**Figure 3:**
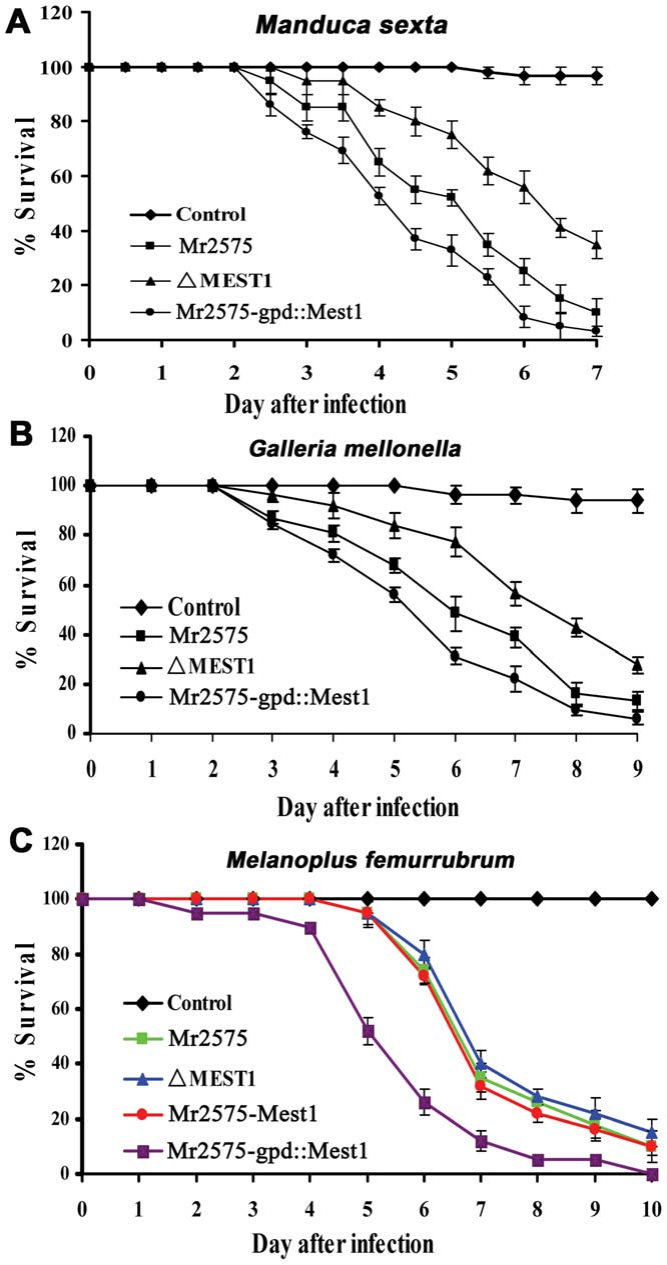
Pathogenicity of *Metarhizium robertsii* strains. (**A**) Survival of *Manduca sexta* larvae following topical application with suspensions of 10^7^ conidia/ml of Mr2575, mutant ΔMest1, or overexpression strain Mr2575-gpd::Mest1 under control of the constitutive *gpd* promoter. (**B**) Survival of *Galleria mellonella* larvae following topical application with suspensions of 10^7^ conidia/ml of Mr2575, mutant ΔMest1, or Mr2575-gpd::Mest1. (**C**) Survival of grasshopper *Melanoplus femurrubrum* following application of 3 µl of 10^8^ conidia/ml of Mr2575, mutant ΔMest1, Mr2575-Mest1 under native promoter control, or Mr2575-gpd::Mest1 per grasshopper pronotum. Control insects were treated with 0.01% Tween-80. Error bars indicate standard errors.

To study the function of *Mest1*, *Mest1* null mutants (ΔMest1), were generated in Mr2575 by homologous replacement (Figure 2 and Table 1). Pathogenicity assays against *Galleria mellonella* and *Manduca sexta* caterpillars revealed a significant reduction in mortality and speed of kill by ΔMest1 relative to wild type *M. robertsii* Mr2575 (Figure 3A and 3B), while virulence against acridid grasshoppers (*Melanoplus femurrubrum*) was unaltered (Figure 3C). To demonstrate that the altered phenotype of ΔMest1 was specifically due to gene inactivation, the *Mest1* gene was reintroduced into ΔMest1 in single copy. Six isolates of the resulting complemented strain (Mest1-Com) infected *Galleria* in an identical fashion as the wild type indicating successful complementation. Overexpression of *Mest1* under control of the constitutive glyceraldehyde-3-phosphate dehydrogenase (*gpd*) promoter (Mr2575-gpd::Mest1) resulted in a significant increase in virulence against both caterpillars (*Manduca* and *Galleria*) and grasshoppers (Figure 3C). Mr2575 transformed with an additional copy of *Mest1* under control of its native promoter increased pathogenicity to lepidopterans but not grasshoppers, suggesting that *Mest1* is not activated by the wild type fungus on grasshoppers.

**Table 1:** List of *Metarhizium* strains used in this study.

Strains	Characterization
Mr2575	Wild type of the generalist strain *M. robertsii* 2575
Ma324	Wild type of the specialist acridid strain *M. acridum* 324
ΔMest1	*M. robertsii* 2575 mutant disrupted in *Mest1*
Mest1-Com	ΔMest1 strain complemented with *Mest1*
Mr2575-Mest1	Mr2575 over-expressing *Mest1* under control of its native promoter
Mr2575-gpd::Mest1	Mr2575 over-expressing *Mest1* under control of the glyceraldehyde-3-phosphate dehydrogenase (*gpd*) promoter
Mr2575-Mest1:GFP	Mr2575 over-expressing a MEST1-EGFP fusion protein under control of the native Mr2575 *Mest1* promoter.
Ma324-Mest1	Ma324 over-expressing the Mr2575 *Mest1* gene under control of the native Mr2575 *Mest1* promoter

**Figure 4:**
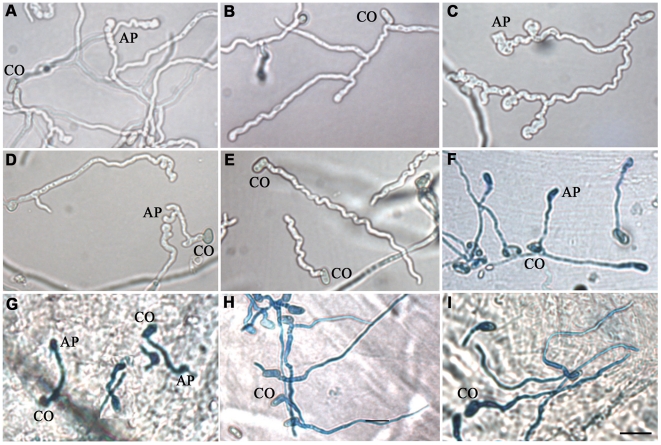
MEST1 regulates appressorial differentiation. Growth of (**A**) wild type *Metarhizium robertsii* Mr2575, (**B**) mutant ΔMest1, (**C**) over-expressing strain Mr2575-gpd::Mest1, (**D**) transgenic strain Ma324-Mest1, and (**E**) wild type *Metarhizium acridum* Ma324 in YE+*Manduca sexta* cuticular lipids. Appressoria formed by transgenic strain Ma324-Mest1 on (**F**) *Galleria mellonella* and (**G**) *M. sexta* cuticles compared to differentiation of wild type Ma324 on (**H**) *G. mellonella* and (**I**) *M. sexta* cuticles 24 h post inoculation. CO, conidia; AP, appressorium. Bar, 10 µm. The fungal hyphae and infection structure were stained by Lactophenol Cotton Blue stain.

To elucidate whether *Mest1* is needed for developmental processes, wild type Mr2575, ΔMest1 and Mest1-Com strains were grown on SDA (Sabouraud dextrose agar) or SDB (Sabouraud dextrose broth) (nutrient rich conditions), in 0.01% YE (Yeast extract) plus *Manduca* cuticular lipids (0.25 mg/ml) and on ground grasshopper or *Manduca* cuticles. There was no significant difference in sporulation, germination and growth rates between *M. robertsii* Mr2575, ΔMest1 and Mest1-Com on SDA or SDB, indicating that *Mest1* does not facilitate these processes in nutrient rich conditions. Germination, germ tube formation and appressorial formation by Mr2575 on intact grasshopper and *Manduca* cuticles were similar (Figure 4). The germination rate of ΔMest1 was significantly (P<0.01) lower than that of wild type *M. robertsii* in 0.01% YE with or without *Manduca* cuticular lipids, and 24 h post-inoculation only 2.7±1.5 and 26.5±2.4% of ΔMest1 germlings had appressoria in YE and YE+lipids, respectively, as compared to 25.5±1.5 (YE) and 81.6±3.2% (YE+lipids) of wild type Mr2575 and 24.7±1.2% (YE) and 80.7±3.5% (YE+lipids) of complemented strain Mest1-Com (Table 2). Furthermore, ΔMest1 appressoria (3.6±0.1 µm×3.9±0.8 µm) were significantly smaller (P<0.01) than those of the wild type (4.1±0.2 µm×12.7±0.4 µm) on insect cuticles (Figure 4A and 4B). Conversely, overexpression of *Mest1* in Mr2575-gpd::Mest1 resulted in multiple lobed appressoria on branched hyphae (Figure 4C).

**Table 2:** Effects of *Manduca sexta* cuticular lipids on conidial germination and appressorial differentiation of *Metarhizium robertsii* strains.

Treatments^a^	Percentage germination (%)						Appressorial formation (%)		
	6 h			10 h			24 h		
	Mr2575	ΔMest1	Mest1-Com	Mr2575	ΔMest1	Mest1-Com	Mr2575	ΔMest1	Mest1-Com
Water	0	0	0	0	0	0	0	0	0
YE	45.5±2.4	22.6±0.8	43.6±3.5	88.5±0.5	61.5±1.5	87.7±1.2	8.6±1.2	0	6.7±1.2
Lipids	32.2±1.8	13.8±1.7	31.5±1.2	64.7±1.0	37.0±1.0	62.8±3.2	25.5±1.5	2.7±1.5	24.7±1.2
YE+lipids	85.2±3.8	42.0±1.0	82.1±0.6	99.5±1.6	82.0±1.0	98.5±1.2	81.6±3.2	26.5±2.4	80.7±3.5

a Cuticular lipids were extracted in dichloromethane. Fungal conidia (2×10^7^ spores ml^−1^) were induced to germinate in 5.5 cm polystyrene petri dishes containing 2 ml of water or 0.01% yeast extract (YE) and/or insect cuticular lipids (0.25 mg/ml). The values are means ± SE.

### Heterologous expression of *Metarhizium robertsii* MEST1 enables *M. acridum* to infect caterpillars

Wild type *M. acridum* Ma324 forms appressoria in locust cuticle lipid extracts and on locust wings [16]. We expressed a 2.7 kb clone encoding *M. robertsii Mest1* under native control in Ma324 and studied its impact on pathogenicity and growth on caterpillar cuticle lipid extracts. Compared to the wild type Ma324, Ma324-Mest1 conidia exhibited significantly faster germination in *Manduca* cuticle lipids with or without YE (P<0.01). Approximately 7% of Ma324-Mest1 germlings produced appressoria in *Manduca* lipids compared to 35% of germlings in lipids+YE (Table 3). In contrast, wild type Ma324 did not form appressoria in either YE or *Manduca* lipid extracts, and <10% of germlings formed appressoria in *Manduca* lipid extracts+YE (Table 3). These appressoria were atypically small (2.8±0.2 µm×3.6±0.7 µm), whereas Ma324-Mest1 formed compound appressoria (3.9±0.3 µm×10.2±0.6 µm) at the end of germ tubes similar to those produced by Mr2575 (Figure 4D). Neither wild type Ma324 nor Ma324-Mest1 formed appressoria in water or YE, even though there was hyphal growth in the latter (Table 3). Thus heterologous expression of *Mest1* by *M. acridum* is not sufficient by itself to trigger differentiation of appressoria.

**Table 3:** Effects of *Manduca sexta* cuticular lipids on conidial germination and appressorial differentiation of *Metarhizium acridum* strains.

Treatments^a^	Percentage germination (%)				Appressorial formation (%)	
	8 h		12 h		24 h	
	Ma324	Ma324-Mest1	Ma324	Ma324-Mest1	Ma324	Ma324-Mest1
Water	0	0	0	0	0	0
YE	23.5±2.0	49.5±2.5	71.0±1.2	86.0±1.6	0	0
Lipids	16.5±1.2	31.8±1.5	35.5±1.9	62.5±2.0	0	6.5±0.7
YE+lipids	61.5±0.5	89.0±2.2	83.5±0.5	97.5±2.5	8.5±1.5	34.5±2.6

a Cuticular lipids were extracted in dichloromethane. Fungal conidia (2×10^7^ spores ml^−1^) were induced to germinate in 5.5-cm polystyrene petri dishes containing 2 ml of water or 0.01% yeast extract (YE) and/or insect cuticular lipids (0.25 mg/ml). The values are means ± SE.

**Figure 5:**
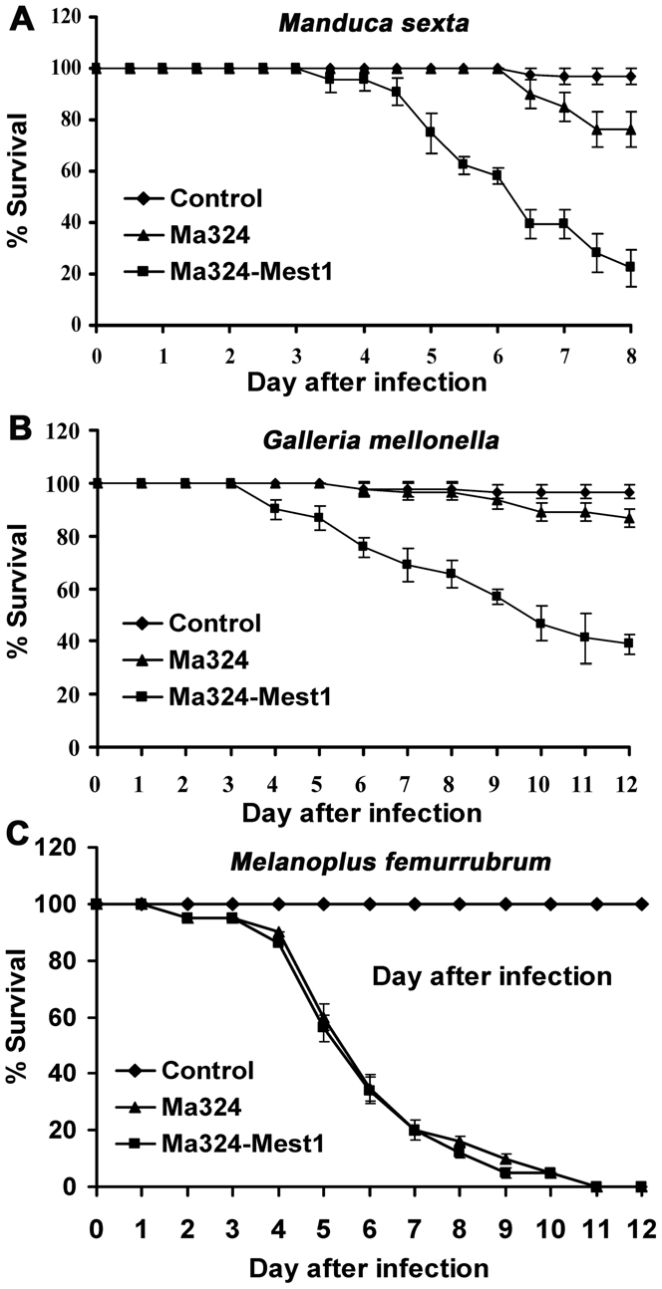
Pathogenicity of *Metarhizium acridum* strains. (**A**) Survival of *Manduca sexta* or (**B**) *Galleria mellonella* larvae following topical application with suspensions of 10^7^ Ma324 or Ma324-Mest1 conidia/ml. (**C**) Survival of *Melanoplus femurrubrum* following application of 3 µl of 10^8^ conidia/ml of Ma324 or Ma324-Mest1 per grasshopper pronotum. Control insects were treated with 0.01% Tween-80. Error bars indicate standard errors.

Unlike wild type Ma324, Ma324-Mest1 produced appressoria within 24 hours of inoculation onto *G. mellonella* and *M. sexta* cuticles (Figure 4F and 4G). Pathogenicity assays showed that Ma324-Mest1 kills *M. sexta* and *G. mellonella* larvae, even though these are very poor hosts for the wild type at the spore dose tested (Figure 5A and 5B). The LT_50_ (time required to kill 50%) values of *M. robertsii* Mr2575 and Ma324-Mest1 against *M. sexta* were similar, being 5.1 days and 6.2 days, respectively. Inoculation of caterpillars with Ma324-Mest1 caused localized melanization (indicating cuticle penetration) and sluggishness similar to Mr2575, whereas caterpillars developed no symptoms with the wild type Ma324 8 days post-inoculation. Ma324-Mest1 was able to complete the full pathogenic life cycle on caterpillars as cadavers quickly became covered in spores. In contrast, wild type Ma324 only produced spores on the cadavers of a preferred acridid host *M. rubrum* (Figure S1). The LT_50_ values of Ma324 (4.9±0.2 d) and Ma324-Mest1 (4.7±0.6 d) against *M. rubrum* were not significantly different (Figure 5C), suggesting that the unknown esterases/lipases used by Ma324 for mobilizing internal nutrients are as efficient as MEST1, but only expressed on its specific hosts.

### Mr2575 expresses MEST1 during nutrient deprivation

**Figure 6:**
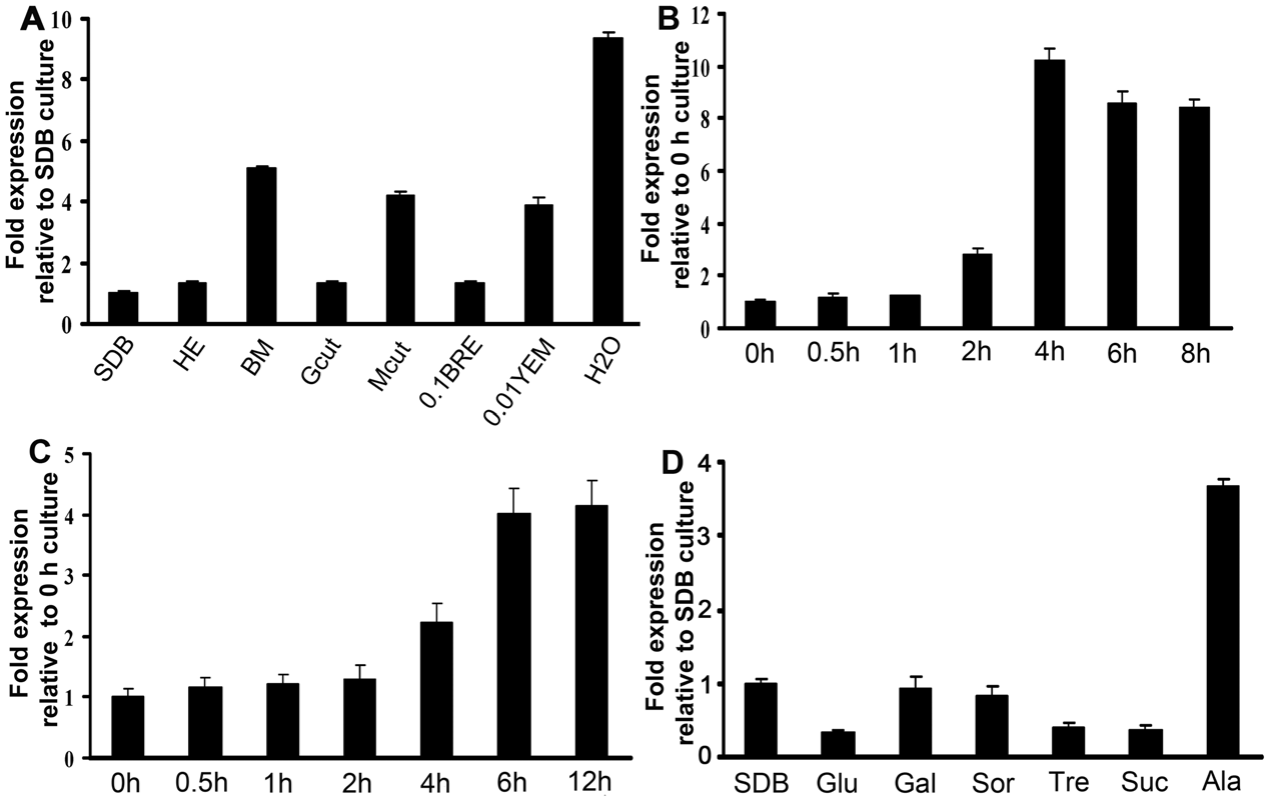
*Mest1* expression in wild type Mr2575, as measured by quantitative real-time RT-PCR. (**A**) Analysis of *Mest1* expression by wild type Mr2575 grown for 6 h in Sabouraud dextrose broth (SDB), cell-free hemolymph (HE), basal medium, 1% grasshopper cuticle (Gcut), 1% *Manduca sexta* cuticle (Mcut), 0.1% bean root exudates (BRE), 0.01% yeast extract medium (YEM) and water. Quantitative RT-PCR time course analyses of *Mest1* expression by wild type *M. robertsii* Mr2575 incubated in (**B**) water for up to 8 h, or (**C**) 1% (w/v) ground *M. sexta* cuticle for up to 12 h. (**D**) Effect of sugars on *Mest1* expression by Mr2575. Quantitative RT-PCR analysis of *Mest1* expression by wild type Mr2575 grown in SDB or basal medium (BM) complemented with 1% glucose (Glu), 1% galactose (Gal), 1% sorbose (Sor), 1% trehalose (Tre), 1% sucrose (Suc) or 1% alanine (Ala). Culture conditions and RNA extraction were as described previously [38]. *gfp* and *tef* were used as reference genes.

Transcript levels of *Mest1* gene and two reference genes *gpd* (glyceraldehyde 3-phosphate dehydrogenase) and *tef* (translation elongation factor 1-α) were measured using SYBR dye technology (Applied Biosystems, CA) and quantitative real-time PCR (qRT-PCR) analysis. Real-time PCR analysis demonstrated stronger expression of *Mest1* by wild type Mr2575 in nutrient-poor media including water, basal medium and 1% *Manduca* cuticle relative to nutrient-rich media such as insect hemolymph or SDB (Figure 6A). We also analyzed *Mest1* expression in time course studies. *Mest1* was activated within 2 h of conidia being incubated in H_2_O but activation took up to 4 h when cultured in 1% ground *Manduca* cuticle medium (Figure 6B and 6C).

Catabolite repression is a common mechanism by which easily available carbon sources decrease the expression of enzymes required for the use of other more complex nutrients such as lipids [23]–[25]. Real-time PCR analysis demonstrated that *Mest1* expression was repressed in Mr2575 when grown on glucose, galactose, sorbose, fructose, trehalose or sucrose as sole carbon sources (Figure 6D). These results suggest that *Mest1* expression occurs when Mr2575 needs to access nutrient reserves. However *Mest1* expression was increased by 1% alanine, a common component of insect cuticles and not by locust cuticle, suggesting that the availability of easily accessible nutrients is not the only controlling factor for *Mest1* expression.

### MEST1 localizes exclusively to lipid droplets

**Figure 7:**
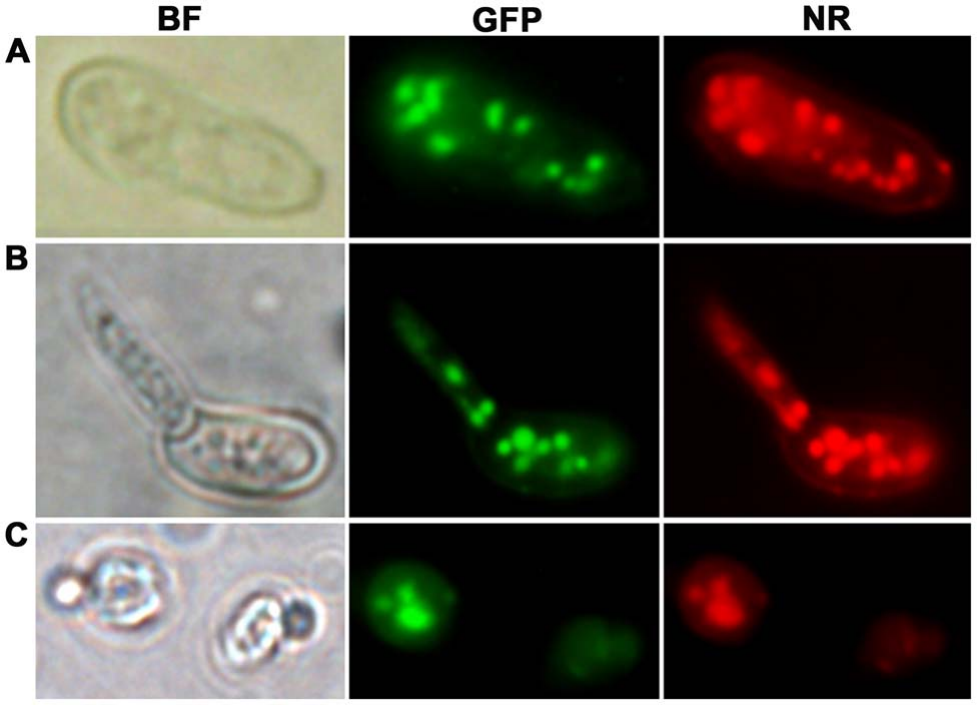
Intracellular localization of MEST1 in *Metarhizium robertsii* Mr2575 and budding yeast *Saccharomyces cerevisiae*. Co-localization of MEST1 and neutral lipids demonstrated by Nile red staining of GFP-MEST1 expressing cells in (**A**) an ungerminated *M. anisopliae* conidium, (**B**) a germinated *M. anisopliae* conidium and (**C**) a budding yeast (*S. cerevisiae*) cell. BF, bright field microscopy. GFP, fluorescence filter. NR, Nile red staining.

To visualize the intracellular targeting of MEST1 *in vivo*, MEST1 tagged at its C-terminus with the green fluorescent protein (GFP) was analyzed by fluorescence microscopy of living Mr2575 cells. We also determined whether lipid droplet localization is a general quality of MEST1 by expressing it in the yeast *S. cerevisiae*, as these lack an endogenous MEST1-like protein. The GFP signal co-localized with lipid droplets stained with the neutral lipid stain Nile red in Mr2575 and the transformed yeast cells, confirming that MEST1 is binding to lipid droplets (Figure 7). No additional diffuse cytoplasmic signal was seen with either GFP or Nile Red.

The expression patterns and the intracellular localization of MEST1 are therefore consistent with the protein playing a part in mobilizing global triacylglycerol storage by acting at the level of lipid droplets.

### MEST1 plays an important role in lipid hydrolysis

**Figure 8:**
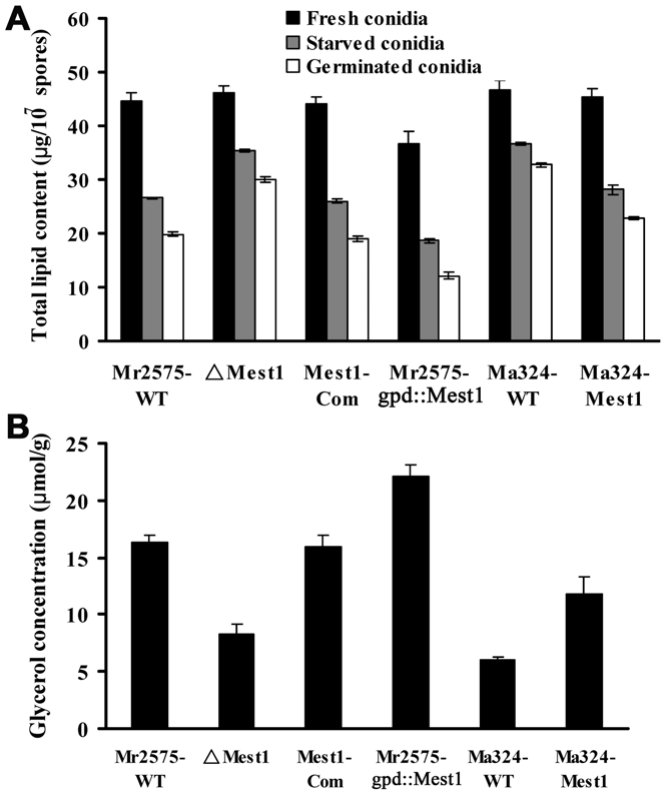
Determination of lipid content. (**A**) Lipid content in conidia freshly harvested from PDA plates (black bars), nutrient-stressed conidia soaked in water for 36 h (grey bars) and conidia germinated in 1% alanine for 12 h (white bars). (**B**) Glycerol content in the six strains grown for 48 h in BM plus 0.25 mg/ml *Manduca* cuticle lipids.

In spite of possessing endogenous nutrient reserves, germination of *M. robertsii* requires external nutrients, albeit these can be at very low levels. When conidia are incubated in water they swell but do not germinate [13]. To test the involvement of MEST1 in lipid metabolism, we compared the lipid content of *M robertsii* Mr2575, ΔMest1, Mest1-Com, Mr2575-gpd::Mest1, *M. acridum* Ma324 and Ma324-Mest1. Except for the reduced lipid content of Mr2575-gpd::Mest1, which constitutively expresses *Mest1*, conidia from the tested strains showed no significant differences in their lipid content, indicating that MEST1 is not required for lipid storage (Figure 8A). As expected, total lipid content in all strains fell significantly (P<0.01) as nutrient reserves were mobilized during nutrient stress (conidia incubated in water) and during germination on 1% alanine. However, germlings of the wild type *M. robertsii* Mr2575 contained only 44.4% of the original lipid present in conidia as compared to 65.2% in the mutant ΔMest1. The complemented strain Mest1-Com had similar lipid content as wild type strain Mr2575. Ma324-Mest1 contained 50.3% of the original lipid as compared to 70% in wild type *M. acridum* Ma324, demonstrating that the transgenic *Mest1* was hydrolyzing lipids in Ma324-Mest1 (Figure 8A).

Triglycerides are degraded into fatty acids and glycerol. Consistent with more rapid hydrolysis of triglycerides, the glycerol content of *M. robertsii* Mr2575 was 1.98-fold higher (P<0.01) than in the disruptant ΔMest1-2575, while the over-expression strain Mr2575-gpd::Mest1 had 1.35-fold higher glycerol than the wild type strain Mr2575 (Figure 8B), suggesting that MEST1 contributes to the generation of turgor pressure in *M. robertsii*. The complemented strain had similar glycerol content as WT Mr2575. Similarly, heterologous expression of *M. robertsii* MEST1 in *M. acridum* Ma324 resulted in a 1.95-fold increase in glycerol content compared to wild type Ma324 (Figure 8B).

To determine if addition of exogenous nutrients overcomes the inability of wild type *M. acridium* to infect lepidopterans, we inoculated *G. mellonella* caterpillars with Ma324 spores suspended in 1% nutrient solutions (SDB, glucose, glycerol, or N-acetylglucosamine), or we topically applied pre-germinated conidia ± exogenous nutrients. Neither exogenous nutrients nor pregermination triggered differentiation of infection structures on insect cuticle, and consequently *M. acridum* was unable to infect caterpillars (Table S2).

### MEST1 hydrolyzes typical esterase substrates

**Figure 9:**
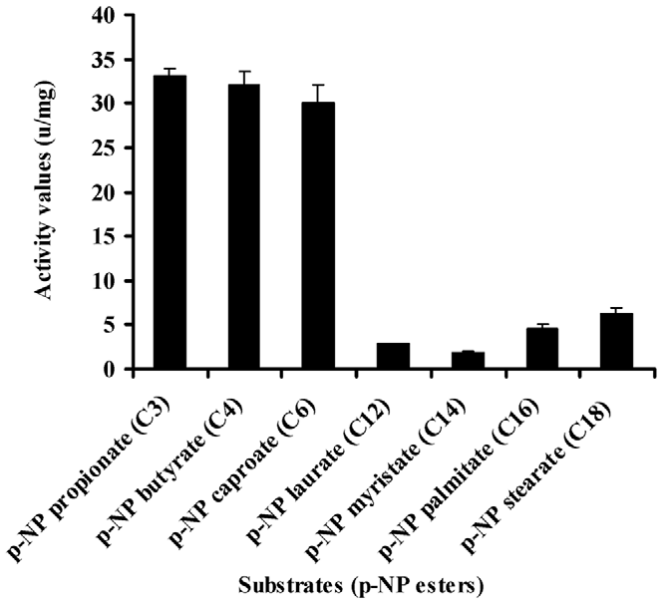
Esterase activity of MEST1 is highest in the presence of short-chain esters (C4–C8). Substrate specificity of MEST1 was determined by hydrolysis of p-nitrophenyl esters with different carbon chain lengths. The activity values are reported as means ± standard errors from three independent assays using crude cell extracts of *E. coli* Rosetta (DE3) expressing MEST1 as compared to extracts from *E. coli* transformed with the corresponding empty vector.

To investigate the substrate specificity of MEST1, we expressed *Mest1* in *E. coli* Rosetta (DE3) cells. SDS-PAGE and western blot analysis confirmed that a novel 47 kDa band in the transformed *E. coli* (ED3) cell lysates was MEST1-(His)6 fusion (Figure 2D and Figure S2). Attempts to purify six-His-tagged MEST1 expressed in *E. coli* Rosetta (DE3) cells failed, which could be because the six-His tag is inaccessible in this protein. Therefore, esterase activity was measured in crude extracts, with similar extracts from *E. coli* Rosetta (DE3) transformed with the corresponding empty vector used as control. The substrate specificity of the expressed MEST1 was determined against p-nitrophenyl esters with different carbon chain-lengths (Figure 9). MEST1 exhibited a marked preference for short-chain fatty acids, with highest activity against p-NP propionate (C3), p-NP butyrate (C4) and p-NP caproate (C6). As is typical for esterases [26], the enzyme was much less active against p-NP esters with longer-chain lengths.

## Discussion

The genus *Metarhizium* provides a novel model system to study evolutionary processes as it includes species such as *M. robertsii* with broad host ranges, as well as *M. majus*, *M. flavoviride* and *M. acridum* that are specific for scarabs, hemipterans and acridids, respectively [5]. *M. acridum* Ma324 in particular has been widely used for locust control. The genetic distinctness of *M. acridum* from generalist strains implies evolutionarily conserved host use patterns. However, being a generalist does not preclude *M. robertsii* strains from showing adaptations to nutrients on frequently encountered hosts. For example, nutrients on Hemiptera (e.g., aphids) are supplemented by insect secretions rich in sugars while beetles carry low levels of nitrogenous nutrients. Consistent with this, many hemipteran-derived lines produce appressoria in glucose medium whereas coleopteran-derived lines do not [27]. Closely related strains show these differences indicating that there are genetic mechanisms allowing rapid adaptation [28].

Lipids are the main nutrient reserve in fungal spores [16], [25]. In addition, lipid bodies are transported to the developing appressoria and degraded to release glycerol, which contributes to the hydrostatic pressure that provides a driving force for mechanical penetration [15]. This process is controlled by a perilipin that surrounds the lipid droplets and is predominantly expressed when *M. robertsii* is engaged in accumulating lipids in nutrient rich conditions. The perilipin layer is broken down during nutrient deprivation which presumably allows esterases/lipases to hydrolyze the lipid [15]. In this paper we show that unlike perilipin, *Mest1* is predominantly expressed when lipids are being broken down. Disrupting *Mest1* (ΔMest1) did not interfere with saprophytic growth of Mr2575 in nutrient rich conditions showing that its function is only important when it is adaptive for the fungus to mobilize endogenous nutrient reserves. Compared to ΔMest1, wild type Mr2575 grown in nutrient poor media or with cuticular lipids germinated faster and the germlings contained less lipid and more glycerol, while overexpression of *Mest1* further boosted germination as well as appressorial differentiation. Intracellular lipid content was also significantly reduced and glycerol content increased in Ma324-Mest1 compared to wild type Ma324. These findings indicate that MEST1 is required by *M. robertsii* for rapid hydrolysis of endogenous energy reserves during germination and for infection structure formation. Lipid droplet enzymes have not been studied in filamentous fungi, but in yeasts all lipid droplet associated proteins are involved in the mobilization of triacylglycerols under conditions of fatty acid starvation [29]. The nutrient poor conditions that induce expression of *Mest1* are a necessary trigger for virulence as *M. robertsii* strain Mr2575 only produces infection structures on cuticle surfaces with low levels of nutrients [13]. However, in ΔMest1 the mobilization of stored lipids was delayed, but not abolished, suggesting the existence of additional enzymes involved in breakdown of lipid droplets.

It seems clear from culturing the Ma324-Mest1 in yeast extract medium, that MEST1 expression is not sufficient by itself to induce appressoria production. Consequently, production of appressoria by Ma324-Mest1 on *M. sexta* and *G. mellonella* cuticles suggests that these have at least some of the inducers required by Ma324 for appressorial differentiation (Figure 2F and 2H). The incidence of infection and the severity of the disease symptoms shown by Ma324-Mest1 were similar to those observed with *M. robertsii* Mr2575, an authentic pathogen of caterpillars. We have therefore shown that inserting a single gene able to mobilize nutrient reserves from a broad spectrum pathogen into a specialist is enough to broaden the latter's host range. This implies that mobilization of nutrient reserves or lack thereof can account for the broad host range of *M. robertsii* and the restricted host range of *M. acridum*, respectively. Restricted host range could therefore be due essentially to biochemical limitations. Addition of exogenous nutrients (SDB, glucose, N-acetylglucosamine, glycerol) or pregermination on rich medium (SDB) did not overcome the inability of *M. acridum* to infect caterpillars. The implication is that intracellular lipid reserves are not only a nutrient source but also a source of chemical signals triggering infection processes. Consistent with this, disrupting the *Mest1* gene in *M. robertsii* (ΔMest1) reduces virulence against caterpillars but not grasshoppers, indicating that the physiological conditions on grasshoppers and caterpillars are different, and there are different mechanisms for linking signals on the host surfaces with differentiation of infection structures. Hydrocarbons comprise over 90% of the wax layer on the surface of grasshoppers, with the balance being composed of wax esters, free fatty acids and triacylglycerides, whereas in larval Lepidoptera, aliphatic alcohols are the most abundant compounds, and triacylglycerides are absent [30]. *M. acridum* extensively hydrolyzes surface lipids and waxes during germination and pre-penetration growth on locust cuticles [31]. A possible explanation for the different responses to grasshopper and caterpillars may be that breakdown products of triglycerides on grasshoppers provide signaling molecules to trigger infection processes by Ma324, but on caterpillars MEST1 provides these signals from stored triglycerides.

As the transgenic *M. acridum* Ma324-Mest1 is able to kill caterpillars, wild type *M. acridum* must have retained most of the genetic machinery required for parasitism of insects outside its natural host range. This is consistent with the developmental processes within *M. anisopliae* and *M. acridum* being very similar, e.g. formation of germ tubes, appressoria, penetration pegs unicellular blastospores, and multi-cellular hyphal bodies that facilitate the infection of target insects, proliferation within haemolymph, and eventual eruption through the host cadaver. Besides novel lineage specific proteins such as MEST1, host recognition may therefore be determined by regulatory controls that allow expression of pathogenicity genes that are not expressed on non-hosts. Expression of *Mest1* under its native *M. robertsii* promoter in *M. acridum* may have broadened host range by bypassing its need for esterases that are regulated by specific locust-related stimuli.

The impact of a single gene on host range suggests that host shifts may have occurred during *Metarhizium* speciation by the acquisition or loss of novel pathogenicity factors. The presence of a MEST1 ortholog in the related *N. haematococca* is consistent with *M. acridum* and *M. robertsii* having inherited MEST1 from a common ancestor. Their patchy distribution could be explained by rapid mutation or multiple lineage specific gene loss events in *M. acridum* and other fungi. An important question is whether utilizing MEST1-like esterases during infection processes is the ancestral state. There are 16 esterases in *M. robertsii* (6 of which are secreted) and 18 in *M. acridum* (6 secreted) [22], and *Metarhizium* spp. express multiple esterases on insect cuticles [32]. It seems likely that Ma324 uses the same esterases for mobilizing lipids as other fungi, and the *Mest1* gene in *M. robertsii* has acquired unique functions in this species as it is clearly highly dispensable. The new functions could potentially have turned the recipient into a novel pathogen or allowed it to infect new hosts. Expression of *Mest1* in Ma324 does not change virulence against its preferred grasshopper host suggesting that acridids do not represent a specialized ecological niche in which a *M. robertsii*-like MEST1 activity is detrimental, and there is no evidence in this study for any conditional benefits in losing MEST1's function. This is consistent with the infection-related functions of MEST1 arising *de novo* in *M. robertsii*.

This study has important safety implications for field applications of *M. acridum* as it shows that Ma324 lacks a gene important for opportunism and this should severely constrain the possibility of host switching to non-target beneficials. In addition, an understanding of how *Mest1* affects fungal responses to hosts, and identification of the signaling cascades involved in regulating the mobilization of nutrient reserves will provide fundamental new insights into the initial steps that are required for the establishment of a compatible interaction between fungi and their hosts.

## Materials and Methods

### Strains and culture conditions

*M. robertsii* Mr2575 and *M. acridum* Ma324 are wild type strains that were obtained from the U.S. Department of Agriculture Entomopathogenic Fungus Collection (ARSEF) in Ithaca, N.Y. Strain Mr2575 can infect caterpillars (*Manduca sexta*), beetles (*Cucurlio caryae*) [33], grasshoppers (*Melanoplus femurrubrum*) and locusts (*Schistocerca gregaria*) [34]. *M. acridum* Ma324 ( = CSIRO FI 485) is the active ingredient of “Green Guard” used for locust control in Australia. In the field it is found exclusively in acridids, and is only infectious to caterpillars in laboratory conditions at very high spore concentrations. Fungal strains were maintained on Potato Dextrose Agar (PDA) at 27°C. Conidia were obtained from 10 day old PDA cultures. For preparation of genomic DNA and RNA, fungal spores were cultured in Sabouraud dextrose broth (SDB) (2×10^6^ conidia/ml) at 27°C.

### *Mest1* gene cloning, disruption, complementation and over-expression

*Mest1* was originally identified as an EST expressed when strain Mr2575 was grown on insect cuticle [17]. The full-length sequence of the *Mest1* cDNA was obtained from the EST using RACE, and a genomic clone was obtained using the DNA Walking Speed Up Kit II (Seegene Inc., Rockville, Maryland, USA). The primers are listed in Supplementary Information, Table S1.

For targeted deletion of *Mest1*, the 5′ and 3′ flanking regions of the *Mest1* ORF were amplified by PCR from Mr2575 genomic DNA, and then subcloned into the *Xba*I and *Spe*I sites of the binary vector pBarGFP [35]. The gene disruption construct (pBarGFP-Mest1) was then transformed into *Agrobacterium tumefaciens* AGL-1 for targeted gene disruption by homologous recombination as described previously [36]. Replacement-specific PCR amplifications of the *Mest1* locus were performed with specific primer pairs (primers are listed in Table S1) that amplify either the wild type or the mutant gene locus.

To revert disruptant ΔMest1, the full-length *Mest1* gene with its native promoter and terminator sequences was amplified by PCR and cloned into the *Xba*I site of pBenGFP [36] to generate complementation vector pBenGFP-Mest1. The complementary strain Mest1-Com was generated by reintroduction of pBenGFP-Mest1 into the disruptant ΔMest1 using *Agrobacterium tumefaciens*-mediated transformation. *M. robertsii* MEST1 was heterologously expressed in specialist *M. acridum* Ma324 using vector pBenGFP-Mest1 to generate Ma324-Mest1. Four putative transformants were chosen and verified for *Mest1* gene expression by RT-PCR (data now shown).

A transformation vector was constructed by amplifying the coding region of *Mest1* from the cDNA clone with primer pairs Mest1F and Mest1R (Table S1) containing a 5′ *Bam*HI site and a 3′ *Eco*RI site plus a 6×His-tag. The resulting PCR fragment was cloned into the pGEM-T/A cloning vector (Promega), and the sequence of the *Mest1* amplicon was confirmed by sequencing. The *Mest1* gene fragment was released with *Bam*H I and *Sma*I, and subcloned into pBarGPE1 [37] downstream of a constitutive *Aspergillus nidulans gpdA* promoter to obtain pBarGPE1-Mest1. The *Mest1* cassette was released by cleavage with *Bgl*II, and then inserted into the *Bgl*II site of pBarGFP to generate pBarGFP-gpdA::Mest1 for *A. tumefaciens*-mediated transformation into Mr2575. The resulting strain was designated as Mr2575-gpd::Mest1. Genomic DNA was extracted from putative transformants for Southern blot analysis as previously described [18]. *Mest1* gene expression in transformants was verified by RT-PCR [38].

### GFP fusion construct and subcellular localization of MEST1

To determine the subcellular localization of MEST1, the promoter region (∼1.5 kb) together with the *Mest1* ORF minus the 3′ TAA stop codon was amplified with primer pairs Mest1F2 and Mest1R2 (Table S1) and inserted into the *Bam*HI and *Eco*RI sites of pBarGPE1 [37] to generate pBarGPE1-Pmest1::Mest1. An enhanced Green Fluorescent gene (eGFP) was amplified from pEGFP (Clontech) with primers gfpF1 and gfpR1 containing a 5′ *Eco*RI site and a 3′ *Xho*I site, and integrated into the *Eco*RI and *Xho*I sites of the plasmid pBarGPE1-Pmest1::Mest1 to generate pBarGPE1-Pmest1::Mest1:GFP. The construct was restricted with *Pml*1 and *Bam*HI, and the released cassette Pmest1::Mest1:GFP was subcloned into the *Bam*HI and *Eco*RV sites of the binary vector pPK2 [39] to generate pBar-Pmest1::Mest1:GFP. The final construct was transformed into wild type Mr2575 using *A. tumefaciens* AGL-1 to generate Mr2575-Mest1:GFP.

We also determined whether MEST1 localizes to lipid droplets in *Saccharomyces cerevisiae*, a fungus unrelated to *Metarhizium* and lacking an endogenous MEST1 protein. The *Mest1* ORF was amplified with primers Mest1yesF and Mest1yesR (Table S1) and the product integrated into the *Eco*RI and *Not*I sites of pYES2 (Invitrogen). The resulting pYES2-Mest1 or the parent plasmid pYES2 (used as a control) were transformed into *S. cerevisiae* strain INVSc1 according to the manufacturer's instructions (Invitrogen). Nile red, (9-diethylamino-5H-benzo [alpha] phenoxazine-5-one) was used to stain intracellular lipid droplets, which were viewed by fluorescence microscopy as previously described [15].

### Reverse transcription and real-time RT-PCR

To monitor *Mest1* expression in different growth conditions, fungal spores were incubated (6 hrs) in 10 ml of fresh SDB, *Manduca sexta* hemolymph [17], basal medium [BM, 0.02% KH_2_PO_4_, 0.01% MgSO_4_ (pH 6)], water or water supplemented with either 0.1% bean root exudate [40]) or 1% insect cuticle as described [38]. Fungal cells were also incubated in BM supplemented with 1% glucose, 1% galactose, 1% sorbose, 1% trehalose, 1% sucrose or 1% alanine. Total RNA was extracted using RNeasy Plant Mini Kit (Qiagen). First strand cDNA was synthesized using Verso cDNA Kit (ABgene) according to manufacturer's instructions. Real-time quantitative reverse transcription PCR (qRT-PCR) reactions were performed using a Quantitative real-time SYBR Green MasterMix Kit (Applied Biosystems) on an Applied Biosystems 7300 real-time instrument and ABI Prism SDS 1.2.2. software. The qPCRs were performed using the following conditions: 50°C for 2 min, then denaturation at 95°C for 10 min followed by 40 cycles of denaturation at 95°C for 20 s, annealing and extension at 60°C for 1 min. The primers used for gene *Mest1* and the reference genes *gpd* and *tef* are listed in Table S1.

### Lipid extraction and quantification

To test the involvement of MEST1 in lipid metabolism, the cell lipid content was quantified by the sulfo-phospho-vanillin method as previously described [15]. A reference standard curve was generated using triolein (Sigma). To determine whether starvation stress induces the hydrolysis of residual stored lipids, conidia from wild type *M. robertsii* Mr2575, mutant ΔMest1, complementary strain Mest1-Com, over-expression strain Mr2575-gpd::Mest1, wild type *M. acridum* Ma324 and transgenic Ma324-Mest1were incubated in H_2_O for 36 h, which causes spores to swell but not germinate, or induced to germinate in basal medium plus 1% alanine (wt/vol) for up to 12 h. The total lipid content of the conidia was assayed as described above.

### Intracellular glycerol measurements

Fungal conidia were inoculated into BM supplemented with 0.25 mg/ml of *Manduca* cuticle lipids [15] for up to 48 h. Cultures were harvested by filtration through Whatman No. 1 filter paper and 0.2 µm Millipore filter units, washed quickly with ice-cold BM, and resuspended in 0.5 M Tris-HCl, pH 7.5. The samples were heated to 95°C for 10 min and cell debris pelleted by centrifugation as described [41]. The glycerol concentration was assayed enzymatically using a glycerol determination kit according to the manufacturer's instructions (Sigma). The data represent the average of three independent experiments.

### Conidial germination and appressorium differentiation

The germination rate of conidia was measured by inoculating 20 µl of spore suspension (2×10^7^ spores ml^−1^) in 5.5 cm polystyrene Petri dishes containing either 2 ml of water, 0.01% yeast exact (YE) and/or insect cuticle lipids (0.25 mg/ml). Three hundred spores from each of three replicates were recorded microscopically to assess germination and appressorial differentiation against the hydrophobic surface of the Petri dish. Appressoria were also induced against locust (*Schistocerca gregaria*) hind wings, *Galleria mellonella* and *Manduca sexta* cuticle as described previously [16].

### Prokaryotic expression and Western blotting of MEST1

For prokaryotic expression of MEST1, the full length cDNA of *Mest1* was cloned by RT-PCR using specific primers Mest1EexF and Mest1EexR (Table S1), restricted with *Eco*RI and *Not*I, and subcloned into the prokaryotic expression vector pET28a at the *Eco*RI and *Not*I sites to form pET28a-Mest1. *E. coli* Rosetta (DE3) cells (Novagen) were transformed with the recombinant expression vector. His–tagged MEST1 production was induced by 0.2 mM IPTG in LB medium for 16 h at 28°C, and cells were lysed with the B-PER Bacterial Protein Extraction Reagent (Thermo Scientific) according to the manufactor's instructions. The His-tagged MEST1 was detected by Western blot analysis using rabbit Anti 6-Histidine Epitope Tag monoclonal antibody and anti-rabbit IgG (Fc), conjugated to alkaline phosphatase (Invitrogen).

### Esterase activity assay

Ester hydrolase activities were determined in *E. coli* Rosetta (DE3) transformed with pET28a-Mest1 or the empty vector pET28a. Cell extract (0.1 ml) containing ∼50 µg of protein and 0.1 ml of a *p*-NP-derivative substrate in 50 mM potassium phosphate buffer (pH 8) was incubated at 37°C. Enzyme activity was spectrophotometrically determined by measuring the liberation of *p*-nitrophenol as previously described [42]. One unit of activity was defined as the amount of enzyme that released 1 µmol of *p*-NP per min per mg of protein under the assay conditions. Substrate specificity towards various *p*-nitrophenyl esters (Sigma-Aldrich) was determined using *p*-NP propionate (C_3_), *p*-NP butyrate (C_4_), *p*-NP caproate (C_6_), *p*-NP laurate (C_12_), *p*-NP myristate (C_14_), *p*-NP palmitate (C_16_), and *p*-NP stearate (C_18_) as substrates.

### Virulence bioassay

The virulence of the wild type *M. robertsii* Mr2575, mutant ΔMest1, complementary strain Mest1-Com, over-expression strain Mr2575-gpd::Mest1, wild type *M. acridum* Ma324 and transgenic strain Ma324-Mest1 were assayed against wild caught 5^th^ instar *Melanoplus femurrubrum* grasshoppers (College Park, Maryland) and newly molted fifth-instar larvae of *Manduca sexta* and *Galleria mellonella* (Carolina Biological supplies). An aliquot of 3 µl of fungal spore suspension was applied to the pronotum of each grasshopper as previously described [43]). Grasshoppers were then placed in clear plastic boxes at 28°C under an 18∶6-h photoperiod in humid conditions (>80%), and supplied daily with fresh wheat seedlings. Each box contained 10 grasshoppers; three containers were used for each dosage of fungus tested (3×10^5^ or 5×10^5^ spores/insect). *Manduca sexta* and *Galleria mellonella* were inoculated by topical immersion in conidial suspensions (1×10^7^ conidia/ml) as previously described [38]. Mortality was recorded every 12 h. After death, cadavers were surface sterilized [35], and incubated in Petri dishes with a sterile wet cotton ball to promote fungal emergence, and thus confirm cause of death. Each treatment was replicated three times with 30 insects per replicate, and the bioassays were repeated twice. LT_50_ values were calculated with the *SPSS* program [44].

### Nucleotide sequence accession numbers

Sequence data reported here have been deposited in the GenBank database under the following accession numbers: *Metarhizium robertsii Mest1* mRNA (HM747114), *Mest1* genomic DNA (HM747115).

## Supporting Information

Figure S1 **Insects infected with** ***Metarhizium*** **strains showing sporulation on cadavers.** (**A**) *Melanoplus femurrubrum* infected with wild type *Metarhizium acridum* Ma324. *Galleria mellonella* infected with transgenic Ma324-Mest1 (**B**) and wild type *Metarhizium robertsii* Mr2575 (**C**). *Manduca sexta* infected with transgenic *M. acridum* Ma324-Mest1 (**D**) and wild type *M. robertsii* Mr2575 (**E**). Dead insects were surface sterilized in 1% bleach for five minutes, rinsed five times with sterile distilled water and placed in sterile Petri dishes containing a wet filter paper to encourage fungal emergence and sporulation.
(TIF)

Figure S2 **Expression of** ***Mest1*** **in** ***E. coli*** **Rosetta (DE3) and yeast** ***Saccharomyces cerevisiae*** **INVSc1.** (**A**) SDS-PAGE of total cellular proteins from *E. coli* Rosetta (DE3) cell lysate harboring empty plasmid pET28a, pET28a-Mest1 without IPTG induction, or pET28a-Mest1 with IPTG induction (4 h). (**B**) SDS-PAGE separation of total cellular proteins from *S. cerevisiae* strain INVSc1 harboring pYES2 or pYES2-Mest1. The size of molecular mass markers is indicated. Arrowheads indicate the target bands of expressed MEST1.
(TIF)

Table S1 **Primer sequences used for PCR amplification.**
(DOC)

Table S2 **Effect of exogenous nutrients on the ability of** ***Metarhizium acridum*** **Ma324 to infect** ***Galleria mellonella*** **.**
(DOC)
