# Supplementary figures and images for: Insertion of an Esterase Gene into a Specific Locust Pathogen (Metarhizium acridum) Enables It to Infect Caterpillars
Source: PLoS Pathog. 2011 Jun 23;7(6):e1002097. doi: 10.1371/journal.ppat.1002097 (PMC3121873; doi:10.1371/journal.ppat.1002097)

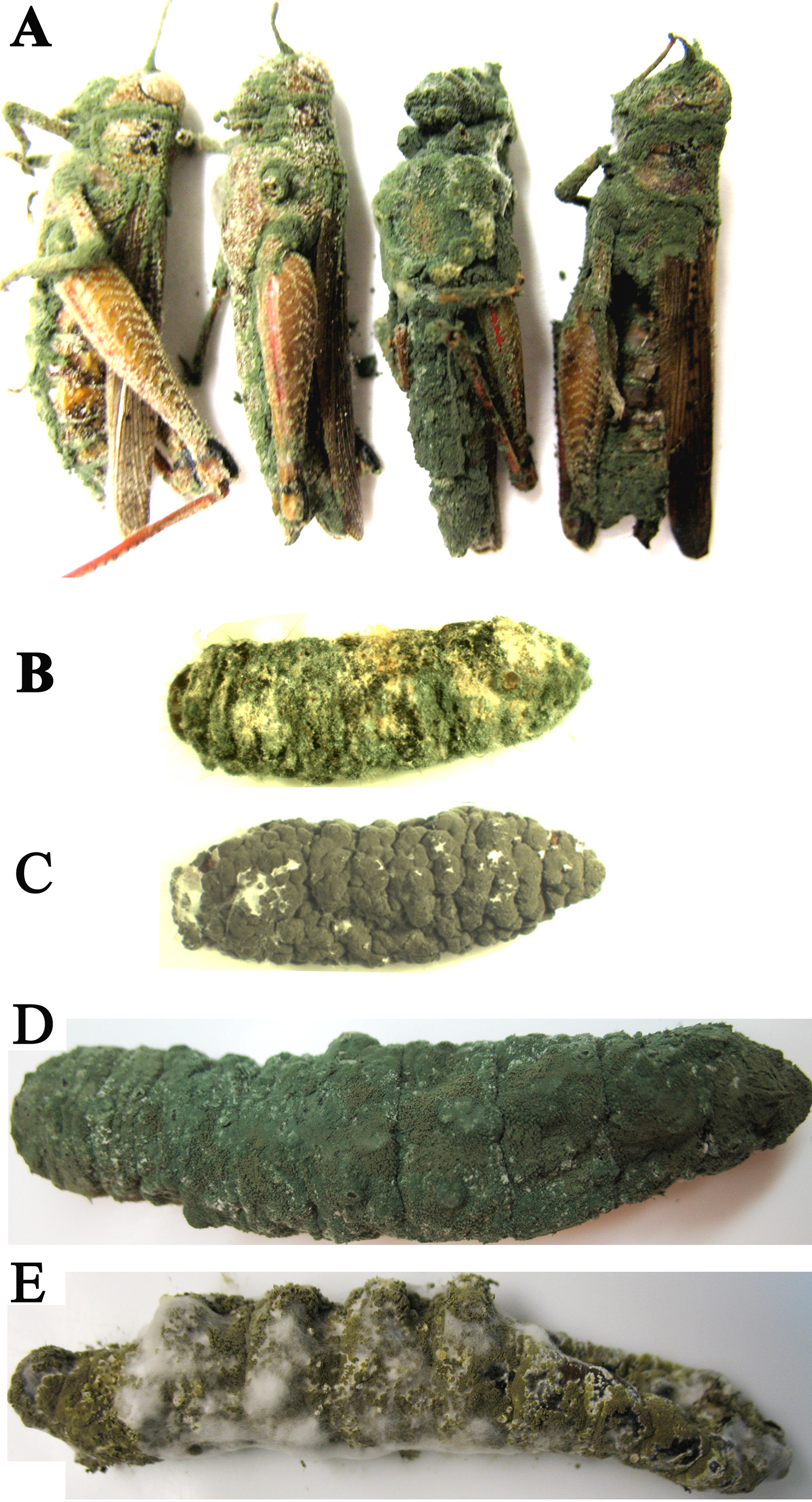

Supplement: Figure S1 — Insects infected with Metarhizium strains showing sporulation on cadavers. (A) Melanoplus femurrubrum infected with wild type Metarhizium acridum Ma324. Galleria mellonella infected with transgenic Ma324-Mest1 (B) and wild type Metarhizium robertsii Mr2575 (C). Manduca sexta infected with transgenic M. acridum Ma324-Mest1 (D) and wild type M. robertsii Mr2575 (E). Dead insects were surface sterilized in 1% bleach for five minutes, rinsed five times with sterile distilled water and placed in sterile Petri dishes containing a wet filter paper to encourage fungal emergence and sporulation. (TIF) [file ppat.1002097.s001.tif]

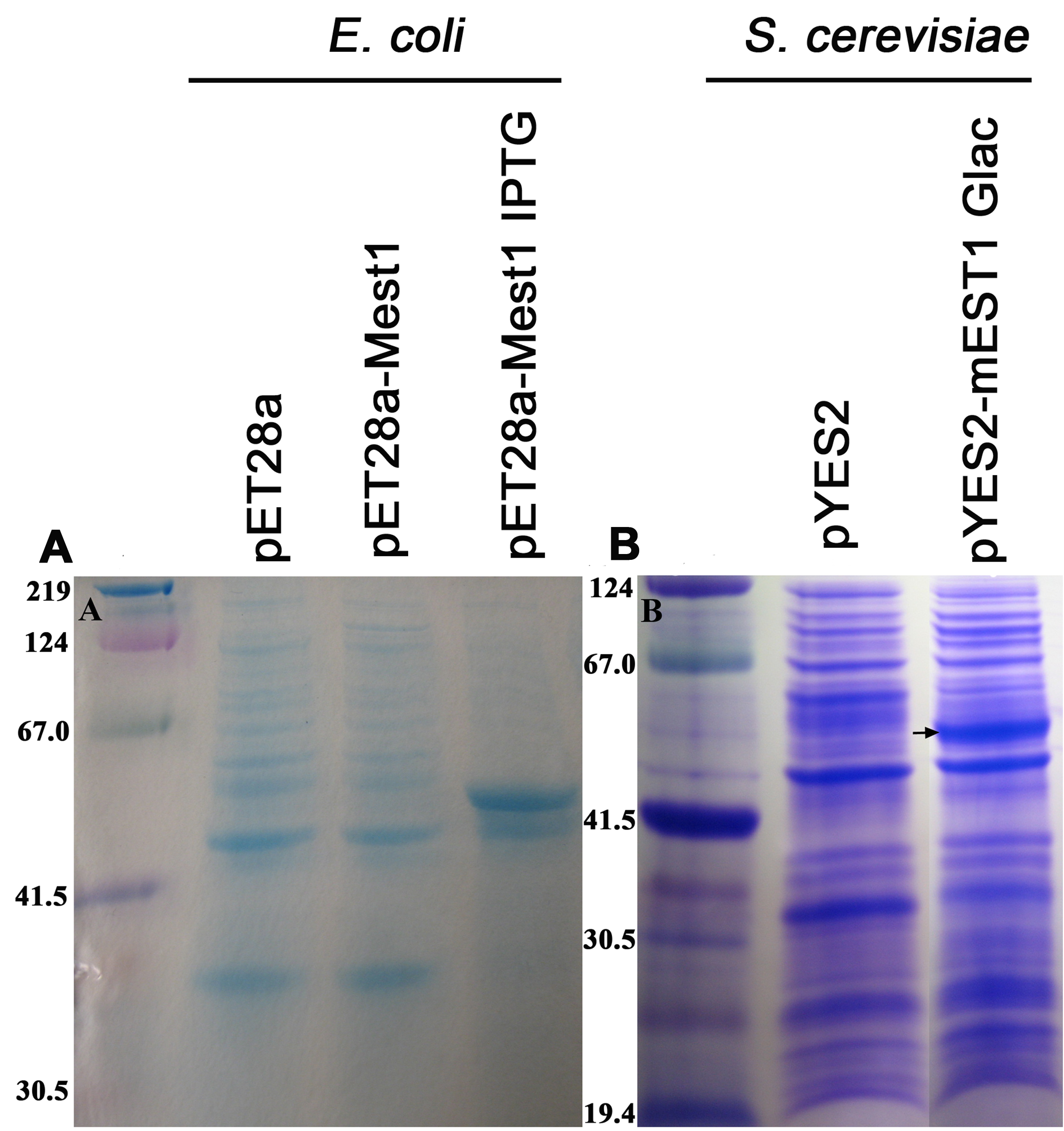

Supplement: Figure S2 — Expression of Mest1 in E. coli Rosetta (DE3) and yeast Saccharomyces cerevisiae INVSc1. (A) SDS-PAGE of total cellular proteins from E. coli Rosetta (DE3) cell lysate harboring empty plasmid pET28a, pET28a-Mest1 without IPTG induction, or pET28a-Mest1 with IPTG induction (4 h). (B) SDS-PAGE separation of total cellular proteins from S. cerevisiae strain INVSc1 harboring pYES2 or pYES2-Mest1. The size of molecular mass markers is indicated. Arrowheads indicate the target bands of expressed MEST1. (TIF) [file ppat.1002097.s002.tif]
