# Supplementary material for: Insertion of an Esterase Gene into a Specific Locust Pathogen (Metarhizium acridum) Enables It to Infect Caterpillars
Source: PLoS Pathog. 2011 Jun 23;7(6):e1002097. doi: 10.1371/journal.ppat.1002097 (PMC3121873; doi:10.1371/journal.ppat.1002097)
Supplement: Table S1 — Primer sequences used for PCR amplification. (DOC) [file ppat.1002097.s003.doc]

**Table S1. Primer sequences used for PCR amplification.**

| **Primer name** | **Primer sequence (5' to 3' end)** | **Restriction sites introduced** |
| --- | --- | --- |
| Mest1F | 5'- CGGGATCCACCACCAACATAACATCCATCA -3' | *Bam*HI |
| Mest1R | 5'-CCCCCGGGTTAATGATGATGATGATGATGGGCAGCTGGGGTCGCTCCGA -3' | *Sma*I |
| Mest1F2 | 5'- CGGGATCCCGCCCAACCGATGATGTCCA -3' | *Bam*HI |
| Mest1R2 | 5'- GGAATTCCGGCAGCTGGGGTCGCTCCGA -3' | *Eco*RI |
| GFPF1 | 5'- CGGAATTCCGATGGTGAGCAAGGGCGAG -3' | *Eco*RI |
| GFPR1 | 5'- CCCTCGAGGGTTACTTGTACAGCTCGTCC -3' | *Xho*I |
| Mest1yesF | 5'- CCGGAATTCCGGACCACCAACATAACATCCATCA -3' | *Eco*RI |
| Mest1yesR | 5'- GCCGCGGCCGCGGCAGCTGGGGTCGCTCCGA -3' | *Not*I |
| Mest1EexF | 5'- CCGGAATTCCGGATGGCCCAGGTCCAAGGC -3' | *Eco*RI |
| Mest1EexR | 5'- GCGCGGCCGCGCTAGGCAGCTGGGGTCGCTC -3' | *Not*I |
| Mest1RTF | 5'- TGGGATACCACGGCTTGA -3' |  |
| Mest1RTR | 5'- AGGATGTTCCCCGTTTAGC -3' |  |
| gpdF | 5'- CGCATCGTCTTCCGCAAC -3' |  |
| gpdR | 5'- TGGGAACACGCATGGACA - 3' |  |
| tefF | 5'- AGGACGACAAGACTCACATC -3' |  |
| tefR | 5'- GTTCAGCGGCTTCCTTCTC -3' |  |
