# Supplementary material for: Insertion of an Esterase Gene into a Specific Locust Pathogen (Metarhizium acridum) Enables It to Infect Caterpillars
Source: PLoS Pathog. 2011 Jun 23;7(6):e1002097. doi: 10.1371/journal.ppat.1002097 (PMC3121873; doi:10.1371/journal.ppat.1002097)
Supplement: Table S2 — Effect of exogenous nutrients on the ability of Metarhizium acridum Ma324 to infect Galleria mellonella . (DOC) [file ppat.1002097.s004.doc]

**Table S2. Effect of exogenous nutrients on the ability of *Metarhizium acridum* Ma324 to infect *Galleria mellonella*.**

| Exogenous nutrient | **Group 1** (spores in 0.05% of Tween-80 solution plus (+) or minus (-) external nutrients)^1^ | | | | |  | **Group 2** (spores were grown at 27°C for 8h in 0.05% of Tween-80 solution plus (+) or minus (-) external nutrients)^1^ | | | | |  | **Group3** (spores were grown at 27°C for 8h in 0.05% of Tween-80 solution plus (+) or minus (-) external nutrients. The external nutrients were then removed and the spores were resuspended in Tween-80 solution )^1^ | | | | |
| --- | --- | --- | --- | --- | --- | --- | --- | --- | --- | --- | --- | --- | --- | --- | --- | --- | --- |
|  | T1^2^ | T2 | T3 | T4 | T5 |  | T1 | T2 | T3 | T4 | T5 |  | T1 | T2 | T3 | T4 | T5 |
| SDB^3^ | - | + | - | - | - |  | - | + | - | - | - |  | - | + | - | - | - |
| 1% glucose | - | - | + | - | - |  | - | - | + | - | - |  | - | - | + | - | - |
| 1% glycerol | - | - | - | + | - |  | - | - | - | + | - |  | - | - | - | + | - |
| 1% NAcGlc^4^ | - | - | - | - | + |  | - | - | - | - | + |  | - | - | - | - | + |
| % Infectivity^5^ | 0 | 0 | 0 | 0 | 0 |  | 0 | 0 | 0 | 0 | 0 |  | 0 | 0 | 0 | 0 | 0 |

Note:

1: Spore concentration was 10^7^ spores per ml in all treatments.

2: T1-T5: different treatments.

3: SDB: Sabouraud dextrose broth

4: NAcGlc: N-Acetyl-glucosamine.

5: Infectivity: percent of insects died due to fungal infections three weeks after fungal inoculation.
